# The perils of cheating

**DOI:** 10.7554/eLife.62222

**Published:** 2020-09-22

**Authors:** M Florencia Camus

**Affiliations:** Department of Genetics, Evolution and Environment, University College LondonLondonUnited Kingdom

**Keywords:** multilevel selection, nutrient availability, cheating, mitochondria, heteroplasmy, metabolism, *C. elegans*

## Abstract

Experiments on mitochondrial DNA in worms highlight that cheating does not always pay off.

**Related research article** Gitschlag B, Tate AT, Patel MR. 2020. Nutrient status shapes selfish mitochondrial genome dynamics across different levels of selection. *eLife* **9**:e56686. doi: 10.7554/eLife.56686

Competition for resources, especially for nutrients, is pervasive in nature and can lead to both cooperation and conflict. This competition can take place at all levels of organisation – from organelles, cells and tissues to whole organisms and populations. Cheating is surprisingly common, with cheaters selfishly using common resources at the expense of others (Maynard Smith and Szathmary, 1997).

Changes in the nutritional environment often lead to competition for resources that can have a dramatic impact on the behaviour and dynamics of different groups (Pereda et al., 2017; Requejo and Camacho, 2011). This raises an important question: how are cooperation and cheating influenced by nutrition? Now, in eLife, Bryan Gitschlag, Ann Tate and Maulik Patel of Vanderbilt University report the results of experiments on the mitochondrial DNA of the nematode *Caenorhabditis elegans* that shed light on this question (Gitschlag et al., 2020).

Mitochondria are organelles that have their own DNA (because, it is thought, they were originally prokaryotic cells that became part of eukaryotic cells as a result of symbiosis). Indeed, mitochondria co-operate with their host cell by providing energy in exchange for a supply of molecular building blocks that are necessary to maintain and replicate mitochondrial DNA. However, a cell can contain different variants of mitochondrial DNA (mtDNA), which creates an environment where distinct mtDNA genomes have to compete to replicate first. Indeed, previous work has shown that cells can contain ‘cheater mitochondria’ that promote their own replication, even if this comes at the expense of the overall fitness of the organism (Klucnika and Ma, 2019).

Gitschlag et al. used genetically modified worms that harboured both wildtype and cheater mtDNA to confirm that the latter is able to replicate more successfully than wildtype mtDNA. Consequently, the offspring contained a higher proportion of cheater mtDNA than their parents (Figure 1). However, cheater mitochondria are not necessarily as efficient as wildtype mitochondria, and Gitschlag et al. found that worms with high levels of cheater mtDNA had decreased levels of mitochondrial function and fertility. So, while cheater DNA is better at invading the next generation, it does so at the expense of the whole organism, and across many generations, cheater mtDNA loses out to wildtype mtDNA at the population level (Figure 1).

**Figure 1.**
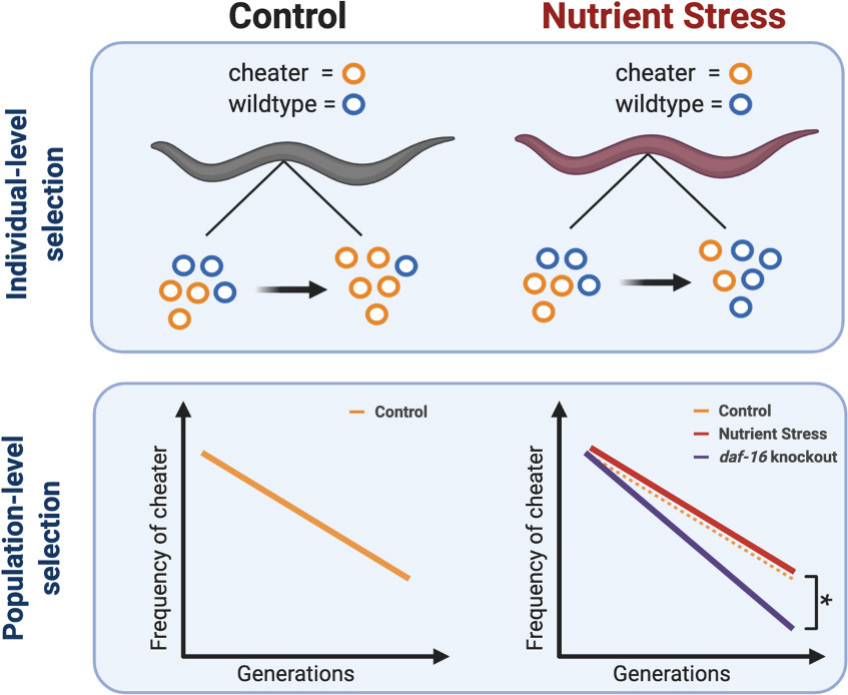
Multilevel selection of cheater mitochondria. Cheater mitochondrial DNA (mtDNA) have a selective advantage that allows it to replicate faster than wildtype mtDNA in individual worms (top left), but this reduces fitness at the population level in successive generations, so the frequency of cheaters decreases with time (bottom left). A lack of nutrients reduces the selective advantage of cheater mtDNA within individual worms (top right), but this has little impact at the population level (bottom right; red and orange lines). However, knocking out a gene called *daf-16*/*foxo* leads to a greater reduction in the frequency of cheaters over time (bottom left; blue line) under conditions of nutrient stress.

To better understand how nutrition – and more specifically nutrient signalling – affected the worms, Gitschlag et al. applied two different strategies: restricting the supply of food to the worms and using genetic techniques to knock out a key nutrient-sensing gene (*daf-16*/*foxo*) in some worms. First, the researchers kept one group of worms on a nutrient-poor diet and a control group on a nutrient-rich diet. They found that worms raised on a restricted diet harboured less cheater mtDNA than those raised on a nutrient-rich diet. Second, without *daf-16*/*foxo*, the selective advantage of cheater mitochondria was also eliminated, and the worms had less cheater mtDNA. This suggests that cheater mtDNA depends on nutrient-sensing genes to proliferate.

Nutrient signalling also affected DNA dynamics at the population level. To study the impact of nutrient deprivation on cheater mtDNA over generations, wildtype and genetically modified worms were kept together in either nutrient-rich or nutrient-poor environments. In the nutrient-poor environments, the proportion of worms carrying cheater mtDNA decreased over generations, albeit at the same rate as in the control group, when *daf-16*/*foxo* was present. On the other hand, worms without *daf-16/foxo* suffered greatly when raised in nutrient-deprived environments, confirming the importance of this gene to cheater mitochondria.

Taken together, these results show that the ability to survive in stressful environments can foster tolerance to cheating, inadvertently prolonging the persistence of cheater genotypes. This suggests that across populations, the genetic response to lack of food can be exploited to partially shield cheater mitochondria from natural selection at the organismal level, which may be useful for understanding diseases associated with mitochondrial dysfunction.
